# Parents, preschoolers, and napping: the development and psychometric properties of two Nap Belief Scales in two independent samples

**DOI:** 10.3389/frsle.2024.1351660

**Published:** 2024-03-19

**Authors:** Adam T. Newton, Graham J. Reid

**Affiliations:** ^1^Department of Psychology, The University of Western Ontario, London, ON, Canada; ^2^Department of Family Medicine and Paediatrics, The University of Western Ontario, London, ON, Canada

**Keywords:** preschool children, child development, sleep, parental perceptions, napping, daytime sleep, psychometrics

## Abstract

**Introduction:**

Most children cease napping between 2 and 5 years old. Little is known about the predictors of this cessation. Parents' sleep-related beliefs aid in understanding children's nighttime sleep behaviors, but few index daytime sleep beliefs.

**Methods:**

Two measures of parents' napping beliefs were developed and evaluated-the Parents' Nap Beliefs Scale (14 items) and the Reasons Children Nap Scale (19 items). Canadian parents of 1–5-year-old children completed these questionnaires and other sleep-related measures in independent pilot (*n* = 201) and replication (*n* = 702) samples. In the replication sample, a subsample of parents also completed 1–3 weeks of daily sleep diaries. The samples were representative of the Canadian population by ethnicity and region.

**Results:**

In both samples, both measures demonstrated strong construct validity, convergent and divergent validity, and internal consistency. The Parents' Nap Beliefs Scale was composed of two factors: (a) Positive Beliefs and (b) Negative Beliefs about napping. The Reasons Children Nap Scale was composed of two higher order factors and five lower order factors: (a) Encouragement Reasons (Child related; Parent related) and (b) Discouragement Reasons (Child prefers not to nap; Child functions well without a nap; Scheduling).

**Discussion:**

Future research should (a) test these scales as longitudinal determinants of children's nap behavior and cessation, (b) evaluate parental Nap Beliefs in non-Western cultures, and (c) adapt these scales for use with childcare providers.

## 1 Introduction

Virtually all 2-year-olds nap, while few 5-year-olds have a daytime nap (Staton et al., 2020). Most preschoolers (2–5 years old) will consolidate their daytime sleep into exclusively nighttime sleep during this period, but little is known about the predictors of this transition. Furthermore, children vary considerably in the frequency and duration of napping during this developmental period. For example, a 5-year longitudinal study of 493 Swiss children found that daily nap durations differed by more than 1.5 h between the 25th and the 75th percentile.

Previous research has identified demographic characteristics (e.g., child age, child sex, ethnicity, and maternal age), perinatal factors (e.g., birth weight and whether birth mother consuming alcohol during pregnancy), childcare arrangements (e.g., child attending daycare), and developmental level as predictors of nap cessation (Crosby et al., 2005; Schwichtenberg et al., 2011; Newton et al., 2023a). However, research to date has largely ignored proximal family influences on children's nap behavior, such as parental beliefs about napping and parental and child preferences (Jones and Ball, 2013). Napping has been associated with several benefits for preschool children (~34–52 months old), including memory consolidation, language acquisition, and emotional/behavioral regulation, generally for habitually napping children (Thorpe et al., 2015; Spencer et al., 2016; Mantua and Spencer, 2017; Spencer, 2021). By comparison, earlier nap cessation has been associated with higher levels of language development, cognitive ability, better emotional regulation, and longer nighttime sleep, and continued napping is associated with longer nighttime sleep onset latency and more frequent night-waking among children older than 2 years old (Dionne et al., 2011; Lam et al., 2011; Werchan and Gomez, 2014; Thorpe et al., 2015; Newton et al., 2023b). See Mantua and Spencer (2017), Thorpe et al. (2015), and Spencer (2021) for more detailed reviews of the association between nap behavior and various outcomes.

Thus, understanding the processes that contribute to nap cessation may have implications for understanding children's development, but psychometrically sound measurements of proximal influences on nap behavior, such as parental beliefs and preferences, are lacking.

We conceptualized children's nap behavior with the socio-ecological model (Jenni and O'Connor, 2005; Grandner, 2014). This model posits that children's transition toward monophasic sleep is influenced by factors at the individual level (e.g., child's development and perinatal factors) the family/social level (e.g., parental beliefs and preferences, parental nap practices, and family functioning), the societal level (e.g., cultural attitudes toward napping and childcare policies), and the interactions between these levels. The present study aimed to enhance the measurement of family/social-level variables by establishing the psychometric properties for two measures of parental nap beliefs, the Parental Nap Beliefs Scale and the Reasons Children Nap Scale. Measurements assessing parents' beliefs about sleep have improved our understanding of young children's nighttime sleep behavior (Sadeh et al., 2007; Coulombe and Reid, 2012). However, no measures exist to index parents' beliefs and preferences about daytime sleep behaviors. Measuring parents' beliefs about naps may improve our understanding of children's nap behaviors. This study provides preliminary and replicated psychometric data for two scales of parents' beliefs around napping for their preschool-aged children.

Some previous research provides templates for quantitively understanding parental nap beliefs. Qualitative data indicate that parents may have specific reasons for encouraging or discouraging their child to nap (Jones and Ball, 2013; Sinclair et al., 2016). In a British qualitative study of parents of preschoolers, parents most often cited naps delaying bedtime as the most common reason for discouraging a nap, while preventing bad tempers/behavior was the most common reason for encouraging a nap (Jones and Ball, 2013). Parents who generally encouraged naps had children who napped for longer and more often than parents who generally discouraged naps. An Australian mixed-method study examined parental attitudes toward napping in early childcare (Sinclair et al., 2016); parents of children 3–6 years old most often (79%) preferred that their children *not* nap regularly while in childcare. Parents who did not want their child to nap regularly in childcare described reasons, such as “nap[ping] interferes with night sleep,” “regular napping is no longer developmentally appropriate,” and “napping is inconsistent with family routine.” Parents who *did* prefer their child to nap regularly described reasons such as “nap[ping] is restorative,” “regular napping is developmentally appropriate,” “naps promote improved mood, behavior, and concentration,” and “nap[ping] is beneficial for family functioning.” Recently, Mindell et al. (2023) surveyed mothers on their perceptions of their 4–36-month-old children's nap behaviors. Nearly all mothers in this sample believed that naps were important (98%), and almost all believed that napping was associated with their child being in a better mood (97%), being easier going (96%), and having fewer tantrums (89%). Furthermore, almost all mothers endorsed that their child napping was beneficial for their own day (94%) and allowed them to do more around the house (92%). These studies highlight two elements for developing questionnaires that can quantify parental beliefs about napping and nap behavior: (a) parents may hold positive and/or negative views about the benefits of napping, and (b) parents see children's napping as impacting the child themselves and the parent/family. Based on this literature and wanting to better understand how parent Nap Beliefs influence nap cessation, we assessed beliefs parents' general beliefs about napping and parents' preferences about encouraging and discouraging naps for their own child.

There are no established theoretical models that outline the beliefs and preferences related to nap behavior for children. However, nap belief measurements have been developed for young adults and reveal three key reasons for napping: (1) appetitive (napping for enjoyment or habitually), (2) restorative (napping in response to subjective fatigue), and (3) prophylactic (napping in preparation for future sleep loss; Milner and Cote, 2009). These reasons are likely related to nap behavior in children. However, this framework requires modification to link conceptually to the reasons preschoolers nap. First, napping is a developmental process among toddlers and preschoolers. That is, 24-h sleep gradually progresses to be consolidated into exclusively nighttime sleep. Second, parents have substantial influence over preschool children's daytime sleep schedules, whereas adults usually dictate their own sleep schedules. Third, parents' beliefs about napping may change as their child ages. A preschool model of Nap Beliefs must account for these processes. Thus, the three-component framework proposed by Milner and Cote (2009) can inform nap belief measurement among children, but it is insufficient. We conceptualized Nap Beliefs among parents of preschoolers using the socio-ecological model to modify the framework proposed by Milner and Cote (2009). Specifically, our theoretical model has an increased focus on (a) multilevel influences (e.g., parental beliefs and scheduling-related factors) and (b) developmental processes (e.g., the normative role of napping and of sleep consolidation). Our model is outlined in the section “Hypothesized Scale Structure.”

### 1.1 Objectives

1. Create two developmentally sensitive scales to index: (a) parents' beliefs about napping, its benefits, and its consequences for preschool-aged children and (b) reasons parents would encourage or discourage naps for their child.
2. Evaluate the scales' psychometric properties (i.e., internal consistency; content, construct, convergent, and divergent validity).
3. Replicate the psychometric properties of these scales in an independent sample.

Two scales were developed: (1) the Parents' Nap Beliefs Scale (which indexes the beliefs about children's napping that parents of preschoolers hold) and (2) the Reasons Children Nap Scale (which indexes the reasons parents would encourage or discourage naps for their child and quantifies parental napping preferences).

### 1.2 Hypothesized scale structure

A rational construct-oriented approach was used in the development of these scales. This approach defines hypothesized constructs *a priori* (Clark and Watson, 1995). The Parents' Nap Beliefs Scale was designed to index parents' beliefs about children's naps, in general. This scale was hypothesized to contain three subscales: (1) Positive Beliefs (napping should be encouraged or has positive effects on sleep and functioning in children), (2) Negative Beliefs (napping should be discouraged or has negative effects on sleep and functioning in children), and (3) Developmental Beliefs (napping is driven primarily by development and that napping will cease on its own). To capture developmental differences, which were expected to influence napping beliefs, parents were asked to consider children about the same age as their child for this scale. This approach has been used in other sleep-related measures for preschool-aged children (Coulombe and Reid, 2012, 2014).

The Reasons Children Nap Scale was designed to index parents' preferences related to their own child's nap behaviors. This scale was hypothesized to contain two higher order factors: Encouragement (reasons parents would urge their child to nap) and Discouragement (reasons parents would dissuade their child from napping). Each of these factors was expected to have two subscales: (1) Child-Related Influences (encourage/discourage napping based on the benefits or consequences for the child) and (2) Parent-/Schedule-Related Influences (encourage/discourage napping based on the benefits or consequences for the parent and family). To capture individual differences, parents were asked to consider their own child for this scale.

Both scales were expected to relate to children's nap duration, napping frequency, and age, the degree to which naps were spontaneous (i.e., child just fell asleep vs. planned naps), and the proportion of the child's sleep during the daytime. These scales were not expected to relate to nighttime sleep problems. Specific hypothesized relations between these scales and measures of convergent and divergent validity are presented in Supplementary Table 1.

## 2 Materials and methods

The two Nap Belief Scales were tested and refined in a pilot sample. The psychometric properties were reevaluated in a replication sample. The procedures and measures for both samples were very similar; any differences are noted in the following sections.

### 2.1 Participants

#### 2.1.1 Pilot sample

Participants were the primary caregivers (i.e., parents) of children (1–5 years old; *N* = 201), residing in Canada. Included were parents who (a) were knowledgeable about daytime and nighttime routines for their child, (b) were comfortable reading in English, and (c) had children without a health condition that interfered with sleep (e.g., autism). To reduce sampling bias, recruitment was targeted to achieve an approximately equal distribution of child age in ½-year increments.

The sample size for this study was based on power guidelines for confirmatory factor analysis (CFA) models which suggest when *N* ≥ 200 and the number of indicators per factor is >3, the power is generally adequate (Kyriazos, 2018).

Most parents were between 30- and 39 years old (58%), white (70%), and employed full-time (62%). About 33% had a bachelor's or other undergraduate degree, and 75% had a family income <CA$100,000. About half of the children were male (52%). See the Supplementary material for detailed sample characteristics (Supplementary Table 3).

#### 2.1.2 Replication sample

As in the pilot sample, participants were the primary caregivers (i.e., parents) of children (1–5 years old; *N* = 702), residing in Canada. The inclusion criteria were identical to the pilot sample. To reduce sampling bias, recruitment was targeted to achieve: (a) an ethnicity distribution approximately equal to the Canadian population and (b) a province-of-residence distribution approximately equal to the Canadian population. Population characteristics were based on the 2016 Canadian Census. These sampling targets were achieved using the quota features on the Qualtrics Survey Management Platform and through targeted emails to potential participants from Qualtrics Panel Services. The replication sample was part of a larger study on the developmental importance of nap cessation among preschool children.

In this sample, most parents were between 30 and 39 years old (67%), white (69%), and employed full-time (52%). About 30% had a bachelor's or other undergraduate degree, and 80% had a family income <CA$100,000. About half of the children were male (54%). See Supplementary material for detailed sample characteristics (Supplementary Table 3).

### 2.2 Procedure

Ethics approval for both samples was granted by the institutional non-medical research ethics board at the University of Western Ontario. For both samples, participants were recruited using Qualtrics Panel Services, and the survey was completed online using the Qualtrics Survey Management Platform. Participants were notified about the study via email by their panel provider. Interested parents completed the screening questionnaire. Then, eligible parents were routed to the letter of information, provided their informed consent, and completed a series of questionnaires. The pilot sample survey took 15 min to complete. The replication sample was part of a larger study on children's napping behavior and took ~30 min to complete. Then, parents were debriefed by providing information on our research objectives and expected results and redirected to their panel provider for compensation. Compensation was known to the participants *a priori*, was determined by Qualtrics, and was unknown to the researchers. This arrangement is standard for projects using Qualtrics Panel Services for recruitment.

In the replication sample, participants were given the option to opt in to complete sleep diaries after completing questionnaires. Participants who consented received email instructions to complete these sleep diaries each day for up to 3 weeks. Participants were asked to complete at least four diaries each week. Participants completing the sleep diaries received compensation directly from the research team: gift cards worth CA$10 for each week completed, plus a bonus incentive for consecutive weeks completed (CA$5 bonus for 2 consecutive weeks completed, CA$10 bonus for 3 consecutive weeks completed).

In both samples, survey quality was assured by (a) using attentional checks (e.g., “select 1 for this item”), (b) straight-lining criteria (participant completion time must be >½ the sample's median completion time), (c) removing participants who provided non-sense textbox responses, and (4) removing participants who provided inconsistent responses throughout the survey (e.g., reported province of residence did not match reported postal code). A complete outline of our data quality approach is available in Supplementary Table 2.

### 2.3 Measures

As the replication sample was part of a larger study on children's nap behavior, only questionnaires related to this report are presented. These measures were identical in both samples unless noted otherwise.

#### 2.3.1 Demographics

Demographic questions were based on items used by Statistics Canada. These items included the parent's age, ethnicity, employment status, relation to the child, and education; the child's age, ethnicity, and sex; and the family's income.

#### 2.3.2 The Parents' Nap Beliefs Scale

This scale indexes parents' beliefs about napping among preschool-aged children. The finalized version contained two subscales: Positive Beliefs and Negative Beliefs about napping. Each item began with a text stem that is customized for each parent, based on the $\frac{1}{2}$-year age range of the child they are reporting on, for example, “Children who are 1–1.5 years old...” This stem guided parents to consider children who were about the same age as their child and account for developmental trends. Parents responded to statements on a 5-point Likert scale from 1, *completely disagree*, to 5, *completely agree*. In the pilot sample, participants completed a preliminary 21-item version of the questionnaire. In the replication sample, participants completed a refined 14-item version of the questionnaire. The development, reliability, and validity of this scale are described later.

#### 2.3.3 The Reasons Children Nap Scale

This scale indexes the extent to which different factors influence parents' decisions to encourage or discourage their child to nap. It contained two higher order factors: Encouragement Reasons and Discouragement Reasons. The stem text for Encouragement Reason items was “I would encourage my child to nap if...,” and for the Discouragement Reason items, the stem was “I would discourage my child from napping if...” Parents responded to statements on a 5-point Likert scale from 1, *Not at all true*, to 5, *Completely true*. In the pilot sample, participants completed a preliminary, 32-item version of this questionnaire. In the replication sample, participants completed a refined 19-item version of this questionnaire. The development, reliability, and validity of this scale are described later.

#### 2.3.4 Validity measures

##### 2.3.4.1 Children's typical daytime sleep behavior

Parents completed a structured retrospective report of their child's typical daytime and nighttime sleep routines, schedule, and behaviors from the General Sleep Inventory (Crosby et al., 2005). The items on typical daytime sleep included the average number of naps during the week and timing and duration of naps. The items on typical nighttime sleep included typical bedtime, sleep onset on weekdays and weekends, and typical wake time. General parent reports are strongly associated with actigraphy (*r* = 0.74; Sadeh, 1994, 1996).

##### 2.3.4.2 Nighttime sleep problems

The Tayside Children's Sleep Questionnaire is a 10-item retrospective parent report of child problems initiating and maintaining nighttime sleep (McGreavey et al., 2005). On the first item, parents report their child's sleep onset latency on a 5-point intensity scale (“How long after going to bed does your child usually fall asleep?”) from “ ≤15 min” to “≥60 min.” On the remaining nine items, parents report on their child's sleep behavior using a 5-point frequency scale from 0 (*sleep behavior never occurs*) to 4 (*sleep behavior happens every night*). This scale demonstrated adequate construct validity (using principal components analysis) and internal consistency (α = 0.85) in its original psychometric evaluation (McGreavey et al., 2005). The total scores from this scale were used to test the divergent validity of the two nap scales.

##### 2.3.4.3 Diary-reported daytime sleep behavior

Parents reported on their children's daily nighttime and daytime sleep duration and quality using sleep diaries. Sleep diaries were only available for the replication sample. For the present study, the sleep diaries assessed (a) the average nap duration across reported days, (b) the proportion of naps that were spontaneous vs. planned over the reported days, (c) the proportion of days with a nap over the reported days, and (d) the average proportion of sleep during daytime over the reported days. Parent-reported sleep diaries are strongly correlated with other measures of sleep (e.g., actigraphy; Hall et al., 2015). Parents were asked to complete at least four out of seven sleep diaries each week and could complete between 1 and 3 weeks of diaries.

### 2.4 Preliminary scale development

#### 2.4.1 Item generation

In total, 58 items (26 for nap beliefs; 32 for reasons children nap) were generated from the adult and pediatric napping literature and from discussions with eight sleep researchers, eight graduate students, and four parents of children 1–5 years old. The three-component framework discussed in the Introduction was used to guide item generation, with adjustments to capture conceptual differences between preschoolers and adults. For example, the Reasons Children Nap Encouragement items “I would encourage my child to nap if... my child told me they wanted a nap” and “I would encourage my child to nap if... I needed a break” are both appetitive and acknowledge that parents have influence over their children's schedules. The Parents' Nap Beliefs Scale item “children my child's age... should have a nap as a normal part of their schedule” acknowledges that napping is normative for many children and that this parent's belief may not be held for older or younger children.

#### 2.4.2 Content validity

Graduate students (*n* = 8) and pediatric sleep researchers and practitioners (*n* = 8) provided feedback on item clarity (from 1, *Not clear at all*, to 5, *Very clear*) and content validity (assignment of which of the hypothesized scales the item belonged to or “none of these”). Any items with a median item clarity rating below four out of five were dropped. One item from the Nap Beliefs Scale was dropped for poor clarity. Items were considered to have poor content validity if (a) fewer than 50% of raters assigned the item to the hypothesized scale or (b) >30% of raters assigned the item to “none of these” (Hinkin and Tracey, 1999). Four items from the Nap Beliefs Scale with poor content validity were dropped. Thus, 21 Nap Beliefs Scale items and 32 Reasons Children Nap Scale items were retained for the pilot sample.

### 2.5 Data analytic plan

Data analyses were conducted in EQS (v. 6.1; CFAs) and SPSS (v. 27; all other analyses).

#### 2.5.1 Factor structure and item reduction

In the pilot sample, two preliminary CFAs were conducted: one for the Nap Beliefs Scale and one for the Reasons Children Nap Scale. First, these models were evaluated using four criteria: (1) standardized residuals below an absolute value of 0.30, (2) factor loadings above 0.70, (3) item-level *R*-squared values above 0.49, and (4) adequate model fit indices [i.e., robust comparative fit index [CFI] ≥ 0.90, robust root mean square error of approximation [RMSEA] ≤ 0.08; Byrne, 2006]. Second, these scales were then adjusted to increase parsimony. Items were selected for removal using four criteria: (1) low item-scale correlations (*r* < 0.30), (2) poor item shape and variability (i.e., high skewness, kurtosis, or low item-variance), (3) standardized residual values above |0.30|, and (4) low factor loadings (<0.70). Furthermore, redundant items were considered for removal based on inter-item correlations and highly correlated error terms. Third, two revised CFAs (one for each scale) were conducted with the reduced items and revised factor structures. The same criteria were applied to evaluate model fit. All CFAs were conducted with maximum likelihood estimation.

In the replication sample, two CFAs were conducted: one for the Nap Beliefs Scale and one for the Reasons Children Nap Scale. These models tested the revised models established in the pilot sample and were evaluated using the four criteria outlined above.

#### 2.5.2 Readability

Using Microsoft Word, Flesch Reading Ease and Flesch-Kincaid Grade Level statistics were generated for the revised versions of each scale, including each scale's instructions and items. Flesch Reading Ease scores between 70 and 80 are considered “fairly easy” to read and scores between 80 and 90 are considered “easy” to read. The Flesch-Kincaid Grade Level is generally equivalent to educational grades in the United States, where grades below 6 are considered “basic” and grades below 8 are considered appropriate for the public (Spadaro et al., 1980).

#### 2.5.3 Internal consistency

Items within identified subscales were averaged to create subscale scores. Then, Cronbach's alphas and inter-item correlations were evaluated. Cronbach's alphas should exceed 0.70, and inter-scale correlations should be <0.85 (Byrne, 2006).

#### 2.5.4 Convergent validity

First, correlations were conducted between the napping scales and children's age (months), children's typical nap duration, and the degree to which naps were spontaneous to test convergent validity. In the replication sample, correlations were also conducted between the typical proportion of child sleep during the daytime. Effect sizes were interpreted using the benchmarks established by Cohen (1988; i.e., *r* = 0.1 = small; *r* = 0.3 = medium; *r* = 0.5 = large). Second, four napping frequency groups were compared: (1) children who had not napped in the past month or napped less than once per week, (2) children who had napped 1–3 days/week, (3) children who had napped 4–5 days/week, and (4) children who had napped 6–7 days/week.^1^ Finally, in the replication sample, correlations between the napping scales and sleep diary reported nap behavior (i.e., proportion of days with a nap, proportion of naps that were spontaneous, average nap duration, and proportion of sleep during the daytime) were assessed. All proportion variables [i.e., the degree to which naps were spontaneous [retrospective reports and sleep diaries], the proportion of sleep during the daytime [retrospective reports and sleep diaries], and the proportion of days with a nap [sleep diaries]] were adjusted using an arcsine transformation prior to analyses.

#### 2.5.5 Divergent validity

In both samples, correlations between the napping scales and nighttime sleep problems were assessed. Effect sizes were interpreted using the benchmarks established by Cohen (1988).

## 3 Results

### 3.1 Preliminary results

In the pilot sample, two participants were missing all items from both Nap Beliefs Scales and were dropped from subsequent analyses. Aside from these cases, missing data were low: 97% of cases were missing no data, 0.5% were missing six values, and 2.5% were missing just one value. Thus, a sample of 199 was utilized for subsequent analyses.

In the replication sample, one participant was missing all items from both Nap Beliefs Scales and was dropped from subsequent analyses. Aside from these cases, missing data were low: 97% of cases were missing no data, 0.28% were missing 7–13 values, 0.86% were missing two values, and 1.86% were missing just one value. Thus, a sample of 701 was utilized for subsequent analyses.

### 3.2 Construct validity

#### 3.2.1 Parents' Nap Beliefs Scale

In the pilot sample, the preliminary CFA was conducted with the 21-item general beliefs scale modeled as a three-factor solution—Positive Beliefs, Negative Beliefs, and Developmentally Related Beliefs. This CFA demonstrated poor model fit (robust CFI = 0.776, robust RMSEA = 0.132). Inspection of the standardized residuals, factor loadings, and *R*-squared values suggested several items that were candidates for removal.

None of the six Developmentally Related items had a factor loading over 0.70 or *R*-squared values over 0.40. As such, the developmental subscale was dropped from subsequent analyses. However, the modification indices suggested a cross-loading of the item “... are too old to nap regularly” on the Negative Beliefs factor. As such, this item was retained and assigned to Negative Beliefs. Additionally, one Positive Beliefs item and one Negative Beliefs item were identified as redundant and were removed.

Following this item reduction, a 14-item Parental Naps Beliefs Scale with a two-factor solution—Positive Beliefs and Negative Beliefs about napping—was tested. This model fit the data well: robust CFI = 0.981, robust RMSEA = 0.052, 90% CI [0.032, 0.071]. This 14-item, 2-factor solution was replicated in the replication sample, robust CFI = 0.960, robust RMSEA = 0.059, 90% CI [0.051, 0.067]. Key scale statistics for both samples are summarized in Tables 1, 2, and inter-item correlations are presented in Supplementary Table 4.

**Table 1:** Item-level descriptive statistics and internal consistencies for the Parents' Nap Beliefs Scale and the Reasons Children Nap Scale in the pilot and replication samples.

	**Pilot sample**		**Replication sample**	
**Item**	***M*** **(*****SD*****)**	α_c_	***M*** **(*****SD*****)**	α_c_
**Parents' Nap Beliefs Scale**				
**Positive Beliefs**	**3.68 (0.98)**	**0.949**	**3.61 (0.89)**	**0.901**
1) Behave better when they nap	3.71 (1.13)		3.94 (1.15)	
2) Should have a nap when they stay up late the night before	3.62 (1.04)		3.73 (1.17)	
3) Should have a nap as a normal part of their schedule	3.58 (1.23)		3.58 (1.23)	
4) Get frustrated more easily when they don't nap	3.76 (1.22)		3.67 (1.25)	
5) Have more meltdowns/tantrums when they don't nap	3.76 (1.19)		3.53 (1.26)	
6) Are better at controlling their emotions when they nap	3.86 (1.12)		3.72 (1.13)	
7) Are more restless when they don't nap	3.69 (1.16)		3.51 (1.22)	
8) Are more easily distracted when they don't nap	3.54 (1.19)		3.29 (1.19)	
9) Listen to their parents better when they nap	3.63 (1.14)		3.54 (1.13)	
**Negative Beliefs**	**3.07 (1.11)**	**0.883**	**3.04 (1.05)**	**0.824**
10) Are too old to nap regularly	2.79 (1.35)		2.72 (1.42)	
11) Do not seem to enjoy napping	3.09 (1.35)		3.09 (1.38)	
12) Do not sleep well at night when they nap that day	3.16 (1.39)		3.09 (1.36)	
13) Will have trouble falling asleep at night when they nap	3.17 (1.35)		3.16 (1.38)	
14) Will resist going to bed if they nap	3.14 (1.30)		3.13 (1.34)	
**Reasons Children Nap Scale**				
**Encouragement**	**3.32 (0.89)**	**0.861**	**3.48 (0.79)**	**0.788**
**Child related**	**3.80 (0.95)**	**0.870**	**3.75 (0.84)**	**0.720**
1) I knew my child would have to stay up late tonight	3.65 (1.22)		4.1 (1.08)	
2) My child had a poor sleep the night before	3.83 (1.12)		3.71 (1.21)	
3) My child told me they wanted a nap	3.92 (1.21)		3.4 (1.33)	
4) Napping was part of my child's routine	3.82 (1.20)		3.71 (1.24)	
5) My child was cranky	3.78 (1.13)		3.84 (1.23)	
**Parent related**	**2.73 (1.30)**	**0.949**	**3.13 (1.22)**	**0.890**
6) I needed free time	2.66 (1.39)		3.14 (1.48)	
7) I needed time to do other things (e.g., chores, relax)	2.72 (1.38)		3.22 (1.35)	
8) The timing was convenient for me	2.75 (1.40)		3.03 (1.39)	
9) I needed a break			3.13 (1.41)	
**Discouragement**	**3.24 (0.96)**	**0.909**	**3.09 (1.01)**	**0.906**
**Child prefers not to nap**	**3.30 (1.15)**	**0.891**	**3.12 (1.21)**	**0.856**
10) My child did not seem to enjoy napping	3.31 (1.30)		3.04 (1.38)	
11) My child refused to nap	3.33 (1.25)		3.18 (1.38)	
12) My child did not want to nap	3.27 (1.23)		3.15 (1.35)	
**Child functions well without a nap**	**3.16 (1.11)**	**0.889**	**2.96 (1.15)**	**0.843**
13) My child slept too much the night before	3.02 (1.28)		2.93 (1.53)	
14) My child got enough sleep the night before	3.06 (1.29)		2.94 (1.37)	
15) My child was in a good mood	3.23 (1.29)		2.94 (1.35)	
16) My child was alert	3.33 (1.26)		3.01 (1.35)	
**Scheduling**	**3.29 (1.07)**	**0.811**	**3.22 (1.14)**	**0.783**
17) I wanted my child to have an earlier bedtime that night	3.18 (1.32)		3.28 (1.39)	
18) There was not enough time for a nap	3.30 (1.21)		3.04 (1.35)	
19) Napping would delay the time my child fell asleep at night	3.38 (1.24)		3.29 (1.35)	

The stem text for Positive Beliefs and Negative Beliefs items is customized for each parent, based on the $\frac{1}{2}$-year age range of the child they are reporting on (e.g., “Children who are 1–1.5 years old...”). The stem text for Encouragement items is “I would encourage my child to nap if...;” the stem text for Discouragement items is “I would discourage my child from napping if...” α = Chronbach's alpha.

**Table 2:** Mean inter-item correlations and factor loadings for the Parents' Nap Beliefs Scale and the Reasons Children Nap Scale in the pilot and replication samples.

	**Pilot sample**		**Replication sample**	
**Item**	**Mean inter-item correlation**	**Factor loading**	**Mean inter-item correlation**	**Factor loading**
**Parents' Nap Beliefs Scale**			
**Positive Beliefs**	**0.673**		**0.502**	
1) Behave better when they nap		0.836		0.732
2) Should have a nap when they stay up late the night before		0.818		0.648
3) Should have a nap as a normal part of their schedule		0.763		0.633
4) Get frustrated more easily when they don't nap		0.806		0.688
5) Have more meltdowns/tantrums when they don't nap		0.825		0.692
6) Are better at controlling their emotions when they nap		0.851		0.765
7) Are more restless when they don't nap		0.787		0.688
8) Are more easily distracted when they don't nap		0.817		0.676
9) Listen to their parents better when they nap		0.803		0.749
**Negative Beliefs**	**0.602**		**0.484**	
10) Are too old to nap regularly		0.767		0.633
11) Do not seem to enjoy napping		0.739		0.649
12) Do not sleep well at night when they nap that day		0.763		0.635
13) Will have trouble falling asleep at night when they nap		0.715		0.654
14) Will resist going to bed if they nap		0.761		0.663
**Reasons Children Nap Scale**				
**Encouragement**	**0.408**		**0.284**	
**Child related**	**0.576**		**0.339**	
1) I knew my child would have to stay up late tonight		0.743		0.613
2) My child had a poor sleep the night before		0.824		0.607
3) My child told me they wanted a nap		0.727		0.568
4) Napping was part of my child's routine		0.724		0.614
5) My child was cranky		0.764		0.542
**Parent related**	**0.822**		**0.669**	
6) I needed free time		0.880		0.823
7) I needed time to do other things (e.g., chores, relax)		0.933		0.770
8) The timing was convenient for me		0.914		0.859
9) I needed a break		0.910		0.864
**Discouragement**	**0.499**		**0.493**	
**Child prefers not to nap**	**0.733**		**0.665**	
10) My child did not seem to enjoy napping		0.813		0.776
11) My child refused to nap		0.904		0.846
12) My child did not want to nap		0.888		0.853
**Child functions well without a nap**	**0.667**		**0.578**	
13) My child slept too much the night before		0.761		0.675
14) My child got enough sleep the night before		0.829		0.795
15) My child was in a good mood		0.863		0.790
16) My child was alert		0.869		0.784
**Scheduling**	**0.591**		**0.546**	
17) I wanted my child to have an earlier bedtime that night		0.741		0.728
18) There was not enough time for a nap		0.769		0.730
19) Napping would delay the time my child fell asleep at night		0.774		0.760

The stem text for Positive Beliefs and Negative Beliefs items is customized for each parent, based on the $\frac{1}{2}$-year age range of the child they are reporting on (e.g., “Children who are 1–1.5 years old...”). The stem text for Encouragement items is “I would encourage my child to nap if...;” the stem text for Discouragement items is “I would discourage my child from napping if...”

#### 3.2.2 The Reasons Children Nap Scale

The preliminary CFA was conducted with the 32 reasons children nap items modeled with two higher order factors and four subscales. This CFA demonstrated poor model fit (robust CFI = 0.751, robust RMSEA = 0.117). Inspection of the standardized residuals, factor loadings, and *R*-squared values suggested several items that were candidates for removal. Across subscales, 13 items were identified as redundant and were removed. Inspection of the modification indices suggested the presence of two child-related discouragement subscales: (1) Child Preference Not to Nap– and (2) Child Functions Well Without a Nap–related reasons.

Following this item reduction and revision of the factor structure, a 19-item scale was tested in a two higher order factor, five lower order factor (i.e., subscales) model: Encourage Napping (Child related, Parent related) and Discourage Napping (Child preference not to nap, Child functions well without a nap, scheduling-related). This model fit the data well: robust CFI = 0.930, robust RMSEA = 0.077, 90% CI [0.066, 0.088]. This factor structure was also replicated in the replication sample, robust CFI = 0.900, robust RMSEA = 0.076, 90% CI [0.071, 0.082]. Key scale statistics are presented in Tables 1, 2.

### 3.3 Readability

The Parents' Nap Beliefs Scale had a Flesch Reading Ease score of 78.0 and a Flesch-Kincaid Grade Level of 5.3. The Reasons Children Nap Scale had a Flesch Reading Ease score of 85.6 and a Flesch-Kincaid Grade Level of 3.4.

### 3.4 Internal consistency

In the pilot sample, all scales and subscales demonstrated strong internal consistency (α_c_ > 0.80). In the replication sample, the Reasons Children Nap Scale's Encouragement second-order factor (α_c_ = 0.79) and Child-related factor (α_c_ = 0.72) demonstrated adequate internal consistency (α_c_ > 0.70). All other scales and subscales demonstrated strong internal consistency (α_c_ > 0.80; see Tables 1, 2). In both samples, no inter-scale correlation exceeded 0.85 (see Table 3). Inter-item correlations are presented in Supplementary Table 5.

**Table 3:** Inter-scale correlations for Nap Belief Scale subscales and the Reasons Children Nap Scale subscales in the pilot and replication samples.

**Subscales**	**(1)**	**(2)**	**(3)**	**(4)**	**(5)**	**(6)**	**(7)**
1) NBS–Positive Beliefs	–	−0.223^**^	0.454^**^	0.402^**^	−0.204^**^	−0.089^*^	−0.038
2) NBS–Negative Beliefs	−0.339^**^	–	−0.037	0.019	0.445^**^	0.468^**^	0.448^**^
3) Encourage–Child Related	0.662^**^	−0.258^**^	–	0.241^**^	0.126^**^	0.077	0.203^**^
4) Encourage–Parent Related	0.338^**^	0.256^**^	0.288^**^	–	0.026	0.294^**^	0.282^**^
5) Discourage–Child Preference	−0.205^**^	0.408^**^	−0.185^**^	0.080	–	0.584^**^	0.577^**^
6) Discourage–Child Functioning	−0.177^*^	0.530^**^	−0.177^*^	0.155^*^	0.587^**^	–	0.710^**^
7) Discourage–Scheduling	−0.082	0.477^**^	−0.108	0.293^**^	0.534^**^	0.682^**^	–

Pilot sample correlations appear below the diagonal and are highlighted in gray. Replication sample correlations appear above the diagonal and are not highlighted. NBS, Nap Beliefs Scale.

^**^p < 0.01. ^*^p < 0.05, adjusted using the false discovery rate.

### 3.5 Convergent validity

Each Nap Beliefs Subscale was correlated with children's age, typical nap duration, and the degree to which naps were spontaneous. The false discovery rate (FDR) was used to adjust for multiple comparisons (Benjamini, 2010). In each sample, 21 correlations were expected and attained (see Table 4). On the Parents' Nap Beliefs Scale, the Positive Beliefs and Negative Beliefs subscales generally correlated with the convergent validity measures in the hypothesized directions with small-to-large (pilot) and medium (replication) effect sizes.

**Table 4:** Correlations between Nap Belief Scale Subscales and continuous convergent validity variables in the pilot and replication samples.

	**Nap Belief Scales**						
	**General beliefs**		**Encourage reasons**		**Discourage reasons**		
**Validity measures**	**Positive**	**Negative**	**Child**	**Parent**	**Child preference**	**Child functioning**	**Parenting/ scheduling**
**Pilot sample—retrospective report**							
Child age	**−0.300** ^**^	**0.417** ^**^	**−0.301** ^**^	0.009	0.153^*^	**0.310** ^**^	0.298^**^
Child's nap duration	**0.373** ^**^	−0.085	**0.307** ^**^	0.267^**^	0.016	−0.026	−0.013
Proportion spontaneous naps	**−0.268** ^**^	0.546 ^**^	**−0.309** ^**^	**0.315** ^**^	0.245^**^	**0.441** ^**^	**0.376** ^**^
Nighttime sleep problems	0.182^*^	0.203^**^	0.224^**^	**0.322** ^**^	0.071	*0.133*	0.194^**^
**Replication sample—retrospective report**							
Child age	**−0.328** ^**^	**0.310** ^**^	−0.114^**^	−0.097^*^	0.169^**^	0.208^**^	0.176^**^
Child's typical nap duration	**0.300** ^**^	**−0.351** ^**^	0.123^**^	0.146^**^	−0.262^**^	−0.200^**^	−0.210^**^
Proportion spontaneous naps	**−0.426** ^**^	**0.417** ^**^	−0.219^**^	−0.073	0.279^**^	**0.336** ^**^	**0.316** ^**^
Proportion of sleep during daytime	**0.411** ^**^	**−0.499** ^**^	0.143^**^	0.243^**^	**−0.336** ^**^	−0.246^**^	−0.261^**^
Nighttime sleep problems	0.087^*^	0.185^**^	0.053	0.085^*^	0.060	0.046	0.086^*^
**Replication sample—sleep diaries**							
Average nap duration	0.520 ^**^	***−0.5*** 92 ^**^	0.240^**^	0.297^**^	−0.299^**^	**−0.366** ^**^	−0.288^**^
Proportion of spontaneous naps	**−0.393** ^**^	**0.446** ^**^	−0.142^*^	−0.218^**^	**0.379** ^**^	**0.426** ^**^	**0.384** ^**^
Proportion of days with naps	0.501^**^	***−0.6*** 18 ^**^	0.218^**^	**0.323** ^**^	**−0.303** ^**^	**−0.384** ^**^	−0.275^**^
Proportion of sleep during daytime	0.528^**^	***−0.6*** 01 ^**^	0.260^**^	**0.347** ^**^	−0.288^**^	**−0.327** ^**^	−0.226^**^

Pilot sample n = 199; Replication sample—retrospective report n = 701; Replication sample—sleep diaries n = 235. To further aid in interpretation, large effect sizes (r ≥ |0.5|) are bolded and underlined; medium effect sizes (r ≥ |0.3|) are bolded; and small effect sizes (r ≥ |0.1|) are italicized.

^*^p < 0.05. ^**^p < 0.01, adjusted using the False Discovery Rate.

On the Reasons Children Nap Scale, encouragement reasons generally correlated with the convergent validity measures in the hypothesized directions with small (pilot) and small-to-medium (replication) effect sizes. In the pilot sample, about half of the discouragement reasons and convergent validity measures were significantly correlated in the hypothesized direction with small or medium effect sizes. In the replication sample, all discouragement subscales correlated with the convergent validity measures in the hypothesized directions with small or medium effect sizes.

**Figure 1:**
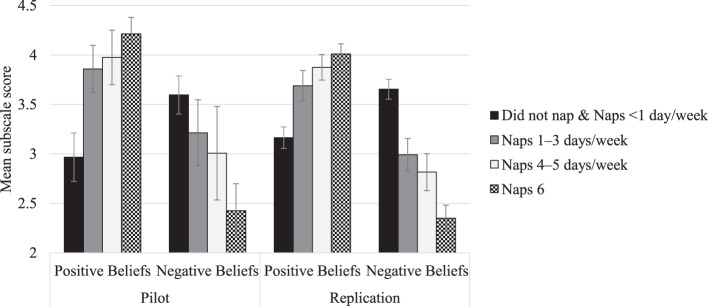
Parental Nap Beliefs and child napping frequency. This figure depicts the relation between mean subscale scores and napping frequency groups for the Positive Beliefs and Negative Beliefs subscales of the Nap Belief Scale. Error bars depict 95% confidence intervals. Supplementary Table 6 reports tests of significance across napping frequency subgroups.

**Figure 2:**
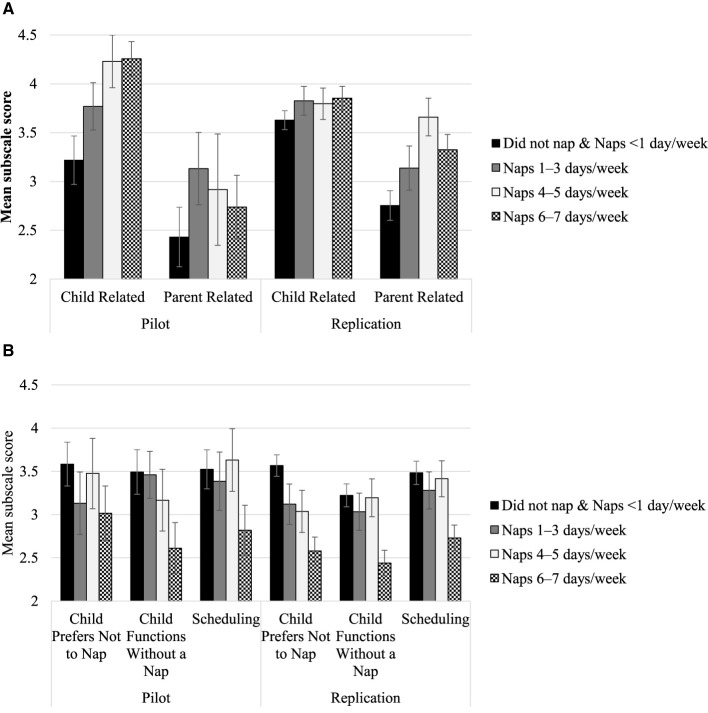
Parental Nap Beliefs and child napping frequency. **(A)** Depicts the relation between the Reasons Children Nap Scale child-related encouragement and parent-related encouragement subscale scores and napping frequency groups. **(B)** Depicts this same relationship but with the child preference-related discouragement (Child Prefers Not to Nap), child functioning-related discouragement (Child Functions Without a Nap), and parenting/scheduling-related discouragement (Scheduling) subscales. Error bars depict 95% Confidence Intervals. Supplementary Table 6 reports tests of significance across napping frequency subgroups.

Five groups of children varying in napping frequency were compared using a one-way ANOVA in each sample. There were significant omnibus differences between napping groups on all subscales for both scales in both samples (*p*-values adjusted using FDR). The general trends were that more frequent napping was related to greater positive beliefs and encouragement reasons, whereas less frequent napping was related to greater negative beliefs and discouragement reasons. In all cases, the “did not nap and naps <1 day/week” group differed significantly from the “naps 6–7 days/week” group at *p* < 0.05. These results are summarized in Figures 1, 2. Full *post hoc* comparisons are presented in Supplementary Table 6.

In the replication sample, each Nap Beliefs Subscale was correlated with sleep diary-derived variables (i.e., average nap duration, the proportion of naps that were spontaneous vs. planned, the proportion of reported days with a nap, and the average proportion of sleep during the daytime). In total, 28 correlations were expected and attained (see Table 4). On the Parents' Nap Beliefs Scale, the Positive Beliefs and Negative Beliefs subscales correlated with the convergent validity measures in the hypothesized directions with medium or large effect sizes. On the Reasons Children Nap Scale, both Encouragement subscales correlated with the convergent validity measures in the hypothesized directions with small or medium effect sizes. On the Reasons Children Nap Scale, all discouragement subscales correlated with the convergent validity measures in the hypothesized directions with small or medium effect sizes.

### 3.6 Divergent validity

Each Nap Beliefs Subscale was correlated with parent-reported nighttime sleep problems. FDR was used to adjust for multiple comparisons. In each sample, seven correlations were expected and attained (see Table 4). On the Parents' Nap Beliefs Scale, both subscales were positively correlated with nighttime problems, with small (pilot) or very small-to-small (replication) effect sizes. On the Reasons Children Nap Scale, both Encouragement and the Scheduling-Related Discouragement subscales were positively correlated with nighttime sleep problems, with very small to medium effect sizes. The child-preference- and child-functioning-related discouragement subscales were not significantly correlated with nighttime sleep problems in either sample.

## 4 Discussion

We have developed two scales to index parents' beliefs about their preschool-aged children's nap behavior. Data from the pilot sample provided promising evidence of the scales' psychometric properties and data from the replication sample, which is representative of the Canadian population, confirmed the scales' psychometric structures, internal consistency, and validity. These scales appear to capture differences in Parents' Nap Beliefs as children age and consolidate sleep.

There were two key differences between our hypothesized scale structures and our revised scales. First, the Developmental Beliefs subscale was removed from the Parents' Nap Beliefs Scale. Developmental beliefs are likely engrained into parents' beliefs about napping. Second, the Discouragement of Napping Child-Related subscale was divided into Child–Preference and Child–Function subscales. This structure may better capture the interaction between parents and children as nap cessation occurs. Specifically, parents' decisions to dissuade their children from napping may be differentially influenced by the child's own preference and children's functioning without a nap.

Our hypothesized convergent validity relations were largely supported. Across both samples and measurement types (i.e., survey and diaries), higher positive beliefs about napping were related to younger child age, longer nap durations, lower proportions of spontaneous naps, greater napping frequency, and higher proportions of sleep during daytime, whereas higher negative beliefs were related to older child age, shorter nap durations (except in the pilot sample), higher proportions of spontaneous naps, lower napping frequency, and lower proportions of sleep during daytime. As hypothesized, the Parents' Nap Beliefs Subscales were not strongly related to parent-reported nighttime sleep problems across samples.

These scales index the beliefs that parents of preschool-aged children hold about napping. Evidence from behavioral genetics studies suggests that napping patterns are more greatly influenced by environmental factors (including parental preferences) than heritable factors, beginning at 2 years old (Touchette et al., 2013). As children begin to consolidate sleep into exclusively nighttime sleep, naps may become increasingly inconsistent (e.g., from napping daily to napping 5 days per week). During this period of transition, parents' beliefs and preferences may be more impactful on children's nap behaviors than before or after the transition to monophasic sleep. The scales presented in this article can be used to understand these beliefs. Furthermore, there is mixed evidence regarding the benefits of napping on behavior and functioning among preschool children (Thorpe et al., 2015). Previous research suggests the benefits of napping may only be present if children are habitual nappers (e.g., napping ≥5 days/week). Finally, these scales integrate well within the socio-ecological model and emphasize the importance of family/social-level influences on preschool children's nap behavior.

We have used the Parents' Nap Beliefs and the Reasons Children Nap Scale as a correlate of children's nap behavior, in the context of other variables (e.g., sociodemographic characteristics, developmental milestones, perinatal factors, nighttime sleep variables, and childcare arrangements; Newton and Reid, 2023). In this study, we used nap characteristics (e.g., nap timing, frequency, duration, spontaneity) to construct four profiles of nap behavior: regular nappers, intermittent nappers, spontaneous nappers, and non-nappers. We then examined the correlates of these profiles. Parents of regular and intermittent nappers tended to have more positive and less negative beliefs on the Parents' Nap Beliefs Scale, compared to non-nappers. Parents of regular nappers tended to have fewer parent-related reasons to discourage napping and more child-related reasons to encourage napping, compared to intermittent nappers. Spontaneous nappers tended to have lesser positive beliefs about children's naps, in general, compared to intermittent nappers (Newton and Reid, 2023). These two scales have been shown to help describe the factors associated with children's nap behaviors in cross-sectional analyses.

### 4.1 Future directions

The pilot and replication samples demonstrated strong psychometric evidence for two scales in diverse samples of Canadian parents. The Nap Beliefs Scale and Reasons Children Nap Scale can be used to understand parental nap preferences across development, across cultures, and as predictors of children's nap behaviors. First, no studies have examined how parents' beliefs about their children's nap behavior change as children age. These scales can be used to understand how parental Nap Beliefs change as children mature and eventually stop napping regularly. Such studies may also utilize a longitudinal methodology to investigate the relations between total 24-h sleep, Parents' Nap Beliefs, and parents' own sleep practices and difficulties (e.g., insomnia).

Second, there is evidence for differences in children's nap behaviors across cultures (Jenni and O'Connor, 2005; Liu et al., 2019). These scales may therefore be useful in exploring cross-cultural differences in beliefs about children's napping. For example, countries such as Italy, Spain, and China tend to prompt more frequent napping (i.e., nap-encouraging cultures). These scales, once appropriately translated, can be used to evaluate between-country differences in parental nap beliefs.

Third, parental beliefs about children's nighttime sleep predict nighttime sleep behavior (Sadeh et al., 2007; Coulombe and Reid, 2012). Parental beliefs around napping may have a similar influence on daytime sleep behavior. These relations should be investigated using longitudinal samples. Importantly, parents often have far less influence over their children's daytime sleep when their child attends childcare. Thus, it would be useful to use adaptions of these scales to quantify childcare providers' beliefs about children's nap behaviors and to investigate whether these beliefs predict nap behaviors within childcare. Our scales may require modifications for use with childcare providers. For example, the Nap Beliefs Scale item “... listen to their parents better when they nap” may change to “... listen to their **educator** better when they nap.” The Reasons Children Nap Scale may also require item modification. For example, there may be a need for a separate subscale that reflects parental wishes that are communicated to the childcare provider (e.g., “I would discourage this child from napping if... **the parent** told me they didn't want the child to nap today”). As such, a modified scale for childcare provider Nap Beliefs and preferences should be developed.

The parental and childcare provider beliefs about napping may interact to predict children's daytime sleep behaviors (Sinclair et al., 2016; Staton et al., 2020). In their Australian mixed-method study, Sinclair et al. (2016) examined parental attitudes toward napping in early childcare. The majority of qualitative reasons parents gave for their napping preferences in this study were related to the child's health and development (92%), including beliefs about the developmental appropriateness of napping. About 10% of parent responses were related to the childcare environment. Within this theme, parents identified that the childcare environment was too noisy or busy, while other parents identified that it was good to have the option for a nap in childcare. Among the parents of 3–6-year-olds, 79% preferred that their child did not nap in childcare. However, nap times in childcare are often used to allow staff to have breaks, clean and organize the room, and engage in professional development (Inglis et al., 2013). Thus, there may be a qualitative conflict between parental preferences and childcare center preferences. Not surprisingly, another study identified marked emotional and behavioral management challenges for childcare staff in centers with mandatory nap times (Pattinson et al., 2014). As such, further investigation of the interaction between parental and childcare provider Nap Beliefs and preferences is required.

### 4.2 Limitations

Our findings should be interpreted with key limitations. First, there were some inconsistencies in our obtained convergent validity correlations, especially for the Reasons Children Nap Scale. These correlations should be replicated in additional samples. Second, our scales and several of our validity measures were obtained via parent-reported questionnaires. As such, some of the associations observed may be due to shared method variance. We sought to mitigate this limitation by including sleep diary measures. This limitation could be further addressed by incorporating actigraphy or videosomnography methodology in future studies. Finally, our convergent and divergent validity analyses were correlational. As such, causality cannot be inferred. Longitudinal studies can be utilized to investigate causality through temporal ordering.

## 5 Conclusion

The Parents' Nap Beliefs Scale and the Reasons Children Nap Scale show strong psychometric properties in a pilot and replication sample of Canadian parents of 1–5-year-old children. Specifically, the scales show strong construct, convergent, and divergent validity and strong internal consistency. These scales are well-suited for use in future preschool napping research. More longitudinal studies are required. Future research should investigate how nap behavior and parents' beliefs about napping change as children age while utilizing actigraphy, in addition to sleep diaries, and monitoring 24-h sleep. Future research can continue to expand the socio-ecological understanding of napping by investigating differences in preschool children's nap behavior by country and culture and the role of childcare providers' beliefs in children's nap behavior. Parents' beliefs about their children's nap behavior aid in understanding children's nap behaviors.

## Data availability statement

The raw data supporting the conclusions of this article will be made available by the authors, without undue reservation.

## Ethics statement

The studies involving humans were approved by Non-Medical Research Ethics Board at the University of Western Ontario. The studies were conducted in accordance with the local legislation and institutional requirements. The participants provided their written informed consent to participate in this study.

## Author contributions

AN: Conceptualization, Writing—original draft, Writing—review & editing, Data curation, Formal analysis, Funding acquisition, Investigation, Methodology, Project administration. GR: Conceptualization, Funding acquisition, Supervision, Writing—review & editing, Methodology, Resources.

## Footnotes

^1^These groupings were based on preliminary analyses in which napping frequency groups from the replication survey (independent variable) were compared using the proportion of reported days with a nap from the sleep diaries (dependent variable) using a one-way analysis of variance (ANOVA; see the Supplemental material).
