# Supplementary material for: Parents, preschoolers, and napping: the development and psychometric properties of two Nap Belief Scales in two independent samples
Source: Front Sleep. 2024 Mar 19;3:1351660. doi: 10.3389/frsle.2024.1351660 (PMC12713905; doi:10.3389/frsle.2024.1351660)
Supplement: Supplementary file 1 [file Data_Sheet_1.docx]

Parents, Preschoolers, and Napping:

The Development and Psychometric Properties of Two Nap Belief Scales in Two Independent Samples

Adam T. Newton & Graham J. Reid

SUPPLEMENTAL MATERIALS

**Table S1.** Hypothesized Relations between Napping Subscales and Convergent Validity Variables

|  | **Nap Belief Scales** | | | | | | |
| --- | --- | --- | --- | --- | --- | --- | --- |
|  | **General Beliefs** | | **Encourage Reasons** | | **Discourage Reasons** | | |
| **Validity Measures** | Positive | Negative | Child | Parent | Child Preference | Child Function | Scheduling |
| **Retrospective Reports** |  |  |  |  |  |  |  |
| Child Age | **-** | **+** | **-** | **-** | **+** | **+** | **+** |
| Child’s Nap Duration | **+** | **-** | **+** | **+** | **-** | **-** | **-** |
| Proportion Spontaneous Naps | **-** | **+** | **-** | **-** | **+** | **+** | **+** |
| Proportion of Sleep During Daytime | **+** | **-** | **+** | **+** | **-** | **-** | **-** |
| Nighttime Sleep Problems | NR | NR | NR | NR | NR | NR | NR |
| **Sleep Diaries** |  |  |  |  |  |  |  |
| Average Nap Duration | **+** | **-** | **+** | **+** | **-** | **-** | **-** |
| Proportion of Spontaneous Naps | **-** | **+** | **-** | **-** | **+** | **+** | **+** |
| Proportion of days with naps | **+** | **-** | **+** | **+** | **-** | **-** | **-** |
| Proportion of sleep during daytime | **+** | **-** | **+** | **+** | **-** | **-** | **-** |

*Note*. “+” represents a predicted positive relation; “-” represents a predicted negative relation; “NR” represents that no relation is predicted.

**Table S2.** Full description of data quality checks applied to the online surveys

| **Criteria** | **Description** | **Action** |
| --- | --- | --- |
| Attentional Checks | 3 attentional checks throughout the survey  e.g., “Select 1 for this item”. Failure is the participant did not select the requested response | Participants who failed 2/3 or more attentional checks were removed from the sample. |
| Straight-lining | Completion time must be greater than half the median completion time | All participants who failed this criterion were removed from the sample. |
| Providing non-sense textbox responses | Participant provides non-sensical textbox responses. Note: participants had the option to skip all textbox responses | All participants who failed this criterion were removed from the sample. |
| Inconsistent location | Participant-provided postal code is outside of participant-provided province | All participants who failed this criterion were removed from the sample. |
| Inconsistent Information | There were 6 inconsistency checks within this criterion:   1. Birthweight well-outside normative ranges for Canadian live births (i.e., low end = 3rd Percentile weight for 31 weeks gestation; high end = 97th percentile for 43 weeks gestation) 2. Height well-outside of normative range for Canadian children 1-5 years (i.e. low end = 3rd percentile for 18 months old and high end = 97th percentile for 6.5 year olds) 3. Weight well-outside of normative range for Canadian children 1-5 years (i.e. low end = 3rd percentile for 18 months old and high end = 97th percentile for 6.5 year olds) 4. Reported “child birth year” does not align with reported “child years old” 5. Reported “child age range” (e.g., 1.5-2 years old) does not grossly aligns with reported “child months old” (+/- 6 months) 6. Sum of reported “older/younger/twin siblings” does not aligns with reported “total number of children” in home | Participants who failed 3/6 or more of these checks were removed rom the sample. |

**Table S3**. Parent, Child, and Family Demographic Characteristics for the Pilot and Replication Samples

|  |  | **Pilot** | **Replication** |
| --- | --- | --- | --- |
| Characteristic | Category | % (*n*) or *M* (*SD*) | % (*n*) or *M* (*SD*) |
| **Parent** |  |  |  |
| Age (years) | Under 21 years | 1.0% (2) | 0.7% (5) |
|  | 21-24 years | 2.0% (4) | 3.0% (21) |
|  | 25-29 years | 11.9% (24) | 12.3% (86) |
|  | 30-34 years | 24.4% (49) | 32.2% (226) |
|  | 35-39 years | 33.8% (68) | 35.0% (246) |
|  | 40-44 years | 17.9% (36) | 11.1% (78) |
|  | 45-49 years | 5.0% (10) | 3.4% (24) |
|  | 50 years or older | 4.0% (8) | 2.1% (15) |
|  |  |  |  |
| Employment status | Employed full-time | 62.2% (125) | 51.6% (362) |
|  | Employed part-time | 13.9% (28) | 9.8% (69) |
|  | On parental leave | 1.0% (2) | 3.1% (22) |
|  | At-home parent | 15.9% (32) | 23.2% (163) |
|  | Student | 1.5% (3) | 1.1% (8) |
|  | Unemployed | 3.5% (7) | 7.5% (53) |
|  | Other (e.g., student with part-time job) | 2.0% (4) | 3.5% (25) |
|  |  |  |  |
| Relation to child | Birth mother | 66.7% (134) | 67.5% (474) |
|  | Birth father | 30.8% (62) | 28.5% (200) |
|  | Other relation (e.g., grandparent) | 2.5% (5) | 4% (28) |
|  |  |  |  |
| Ethnicity^1^ | White | 70.1% (141) | 68.7% (482) |
|  | Chinese | 7.5% (15) | 6.1% (43) |
|  | South Asian (e.g., Punjabi, Sri Lankan) | 5.5% (11) | 1.7% (12) |
|  | Indigenous Persons (e.g., First Nations) | 4.0% (8) | 2.8% (20) |
|  | Black | 3.5% (7) | 5.4% (38) |
|  | Southeast Asian (e.g., Cambodian) | 3.5% (7) |  |
|  | Arab/West Asian (e.g., Armenian, Iranian) | 2.5% (5) | 1.9% (13) |
|  | Filipino | 2.5% (5) | 3.1% (22) |
|  | Latin American | 2.0% (4) | 3.6% (25) |
|  | Korean | 1.5% (3) | 0.4% (3) |
|  | Japanese | 1.0% (2) | 1.3% (9) |
|  | Other ethnicities | 1.0% (2) | 2.0% (14) |
|  |  |  |  |
| Education level | Some high-school or lower | 1.5% (3) | 3.1% (22) |
|  | High school graduate/GED | 15.4% (31) | 12.3% (86) |
|  | Some post-secondary | 12.4% (25) | 18.3% (128) |
|  | Diploma from college/nursing school | 20.9% (42) | 18.0% (127) |
|  | Undergraduate degree | 32.3% (65) | 29.6% (208) |
|  | Masters, professional degree (e.g., MD), or earned doctorate (e.g., PhD) | 16.9% (34) | 18.3% (129) |
|  | Prefer not to answer | 0.5% (1) | 0.0% (0) |
| **Child** |  |  |  |
| Age | 1-year-old | 18.9% (38) | 15.0% (105) |
|  | 2-years-old | 22.4% (45) | 20.7% (145) |
|  | 3-years-old | 21.4% (43) | 17.8% (125) |
|  | 4-years-old | 20.4% (41) | 20.8% (146) |
|  | 5-years-old | 16.9% (34) | 25.8% (181) |
|  |  |  |  |
| Sex | Male | 52.5% (105) | 53.8% (378) |
|  | Female | 47.3% (95) | 45.6% (320) |
|  | Prefer not to answer | 0.0% (0) | 0.5% (4) |
| **Family** |  |  |  |
| Income | < $40,000 | 15.4% (31) | 19.1% (134) |
|  | $40,000 to $59,999 | 14.9% (30) | 11.7% (82) |
|  | $60,000 to $79,999 | 14.9% (30) | 15.7% (110) |
|  | $80,000 to $99,999 | 22.9% (46) | 29.7% (209) |
|  | ≥ $100,000 | 26.9% (54) | 20.4% (143) |
|  | Prefer not to answer | 5.0% (10) | 3.4% (24) |

*Note.* ^1^ Participants could select more than one category.

**Table S4a.** Inter-item Pearson correlations for the Parents’ Nap Beliefs Scale in the Pilot Sample

|  | Positive Beliefs Items | | | | | | | | |  | Negative Beliefs Items | | | | |
| --- | --- | --- | --- | --- | --- | --- | --- | --- | --- | --- | --- | --- | --- | --- | --- |
|  | **(1)** behave better when they nap | **(2)** should have a nap when they stay up late the night before | **(3)** should have a nap as a normal part of their schedule | **(4)**  get frustrated more easily when they don-t nap | **(5)**  have more meltdowns tantrums when they don’t nap | **(6)**  are better at controlling their emotions when they nap | **(7)**  are more restless when they don’t nap | **(8)**  are more easily distracted when they don’t nap | **(9)**  listen to their parents better when they nap |  | **(10)**  are too old to nap regularly | **(11)**  do not seem to enjoy napping | **(12)**  do not sleep well at night when they nap that day | **(13)**  will have trouble falling asleep at night when they nap | **(14)** will resist going to bed if they nap |
| (1) |  | .66*** | .59*** | .69*** | .69*** | .75*** | .64*** | .69*** | .82*** |  | -0.32*** | .23** | -.28*** | -.24*** | -.25*** |
| (2) |  |  | .60*** | .64*** | .67*** | .63*** | .62*** | .66*** | .63*** |  | -.23** | -.17* | -.16* | -.16* | -.22** |
| (3) |  |  |  | .70*** | .68*** | .63*** | .59*** | .65*** | .55*** |  | -.40*** | -.29*** | -.40*** | .39*** | -.35*** |
| (4) |  |  |  |  | .80*** | .71*** | .70*** | .75*** | .63*** |  | .29*** | -.20** | -.24*** | -.25*** | -.28*** |
| (5) |  |  |  |  |  | .74*** | .75*** | .78*** | .65*** |  | -.25*** | .25*** | .23** | -.27*** | -.27*** |
| (6) |  |  |  |  |  |  | .66*** | .72*** | .72*** |  | -.27*** | -.17** | -.23** | -.26*** | -.26*** |
| (7) |  |  |  |  |  |  |  | .71*** | .60*** |  | -.22** | -.11 | -.13 | -.19** | -.18** |
| (8) |  |  |  |  |  |  |  |  | .66*** |  | -.24*** | -.11 | -.21** | -.20** | -.19** |
| (9) |  |  |  |  |  |  |  |  |  |  | -.25*** | -.13 | -.21** | -.16* | -.17* |
| (10) |  |  |  |  |  |  |  |  |  |  |  | .58*** | .65*** | .51*** | .55*** |
| (11) |  |  |  |  |  |  |  |  |  |  |  |  | .56*** | .46*** | .54*** |
| (12) |  |  |  |  |  |  |  |  |  |  |  |  |  | .68*** | .66*** |
| (13) |  |  |  |  |  |  |  |  |  |  |  |  |  |  | .80*** |

**Note.* The stem text for these items is “Children of my child’s age”;

* *p* < .05, ** *p* < .01, *** *p* < .001. r’s greater than |.16| are significant at *p* < .05.

**Table S4b.** Inter-item Pearson correlations for the Parents’ Nap Beliefs Scale in the Replication Sample

|  | Positive Beliefs Items | | | | | | | | |  | Negative Beliefs Items | | | | |
| --- | --- | --- | --- | --- | --- | --- | --- | --- | --- | --- | --- | --- | --- | --- | --- |
|  | **(1)** behave better when they nap | **(2)** should have a nap when they stay up late the night before | **(3)** should have a nap as a normal part of their schedule | **(4)**  get frustrated more easily when they don-t nap | **(5)**  have more meltdowns tantrums when they don’t nap | **(6)**  are better at controlling their emotions when they nap | **(7)**  are more restless when they don’t nap | **(8)**  are more easily distracted when they don’t nap | **(9)**  listen to their parents better when they nap |  | **(10)**  are too old to nap regularly | **(11)**  do not seem to enjoy napping | **(12)**  do not sleep well at night when they nap that day | **(13)**  will have trouble falling asleep at night when they nap | **(14)** will resist going to bed if they nap |
| (1) |  | 0.46*** | 0.52*** | 0.50*** | 0.48*** | 0.55*** | 0.42*** | 0.41*** | 0.55*** |  | -0.24*** | -0.20*** | -0.07 | -0.21*** | -0.19*** |
| (2) |  |  | 0.45*** | 0.38*** | 0.37*** | 0.37*** | 0.34*** | 0.31*** | 0.39*** |  | -0.21*** | -0.16*** | -0.09* | -0.15*** | -0.19*** |
| (3) |  |  |  | 0.49*** | 0.48*** | 0.53*** | 0.49*** | 0.42*** | 0.48*** |  | -0.32*** | -0.20*** | -0.15*** | -0.26*** | -0.23*** |
| (4) |  |  |  |  | 0.67*** | 0.58*** | 0.58*** | 0.51*** | 0.54*** |  | -0.24*** | -0.10* | -0.08* | -0.16*** | -0.18*** |
| (5) |  |  |  |  |  | 0.61*** | 0.61*** | 0.56*** | 0.56*** |  | -0.19*** | -0.10* | -0.06 | -0.14*** | -0.10* |
| (6) |  |  |  |  |  |  | 0.55*** | 0.52*** | 0.64*** |  | -0.21*** | -0.12** | -0.06 | -0.18*** | -0.11** |
| (7) |  |  |  |  |  |  |  | 0.54*** | 0.53*** |  | -0.14*** | -0.07 | -0.03 | -0.09* | -0.06 |
| (8) |  |  |  |  |  |  |  |  | 0.50*** |  | -0.08* | -0.03 | 0.09* | 0.01 | 0.04 |
| (9) |  |  |  |  |  |  |  |  |  |  | -0.14*** | -0.07 | -0.03 | -0.12** | -0.08* |
| (10) |  |  |  |  |  |  |  |  |  |  |  | 0.43*** | 0.38*** | 0.44*** | 0.42*** |
| (11) |  |  |  |  |  |  |  |  |  |  |  |  | 0.39*** | 0.44*** | 0.44*** |
| (12) |  |  |  |  |  |  |  |  |  |  |  |  |  | 0.63*** | 0.58*** |
| (13) |  |  |  |  |  |  |  |  |  |  |  |  |  |  | 0.68*** |

**Note.* The stem text for these items is “Children of my child’s age”;

* *p* < .05, ** *p* < .01, *** *p* < .001. *r*’s greater than |.075| are significant at *p* < .05.

**Table S5a.** Inter-item Pearson correlations for the Reasons Children Nap Scale in the Pilot Sample

|  | Encouragement Reasons | | | | | | | | | |  | Discouragement Reasons | | | | | | | | | | | |
| --- | --- | --- | --- | --- | --- | --- | --- | --- | --- | --- | --- | --- | --- | --- | --- | --- | --- | --- | --- | --- | --- | --- | --- |
|  | ***Child-related*** | | | | |  | ***Parent-related*** | | | |  | ***Child-Preference*** | | |  | ***Child-Function*** | | | |  | ***Scheduling*** | | |
|  | **(1)**  I knew my child would have to stay up late tonight | **(2)**  My child had poor sleep the night before | **(3)**  My child told me they wanted a nap | **(4)** Napping was part of my child’s routine | **(5)**  My child was cranky |  | **(6)**  I needed free time | **(7)**  I needed time to do other things e.g., chores, relax | **(8)**  The timing worked for me | **(9)**  I needed a break |  | **(10)**  My child did not seem to enjoy napping | **(11)**  My child refused to nap | **(12)**  My child did not want to nap |  | **(13)**  My child slept too much the night before | **(14)**  My child got enough sleep the night before | **(15)**  My child was in a good mood | **(16)**  My child was alert |  | **(17)**  I wanted my child to have an earlier bedtime that night | **(18)** There was not enough time for a nap | **(19)** Napping would delay the time my child fell asleep at night |
| (1) |  | .71*** | .50*** | .53*** | .51*** |  | .15* | .24*** | .24*** | .25*** |  | -.12 | -.16* | -.15 |  | .01 | -.13 | -.12 | -.06 |  | .03 | -.01 | -.04 |
| (2) |  |  | .60*** | .56*** | .60*** |  | .19** | .27*** | .27*** | .28*** |  | -.09 | -.17* | -.12 |  | -.01 | -.09 | -.12 | -.09 |  | -.10 | -.12 | -.07 |
| (3) |  |  |  | .50*** | .60*** |  | .08 | .16* | .13 | .12 |  | -.07 | -.08 | -.03 |  | -.10 | -.13 | -.09 | .09 |  | -.05 | -.05 | -.07 |
| (4) |  |  |  |  | .56*** |  | .19** | .244*** | .26*** | .24*** |  | -.20** | -.21** | -.18* |  | -.29*** | -.21** | .23** | .18* |  | -.24*** | -.20** | -.21** |
| (5) |  |  |  |  |  |  | .17* | .28*** | .26*** | .29*** |  | -.13 | -.16* | -.11 |  | -.16* | -.11 | -.18* | -.14 |  | -.05 | -.09 | -.05 |
| (6) |  |  |  |  |  |  |  | .83*** | .79*** | .78*** |  | .10 | .06 | .04 |  | .05 | .17* | .15* | .08 |  | .26*** | .25*** | .15* |
| (7) |  |  |  |  |  |  |  |  | .85*** | .85*** |  | .09 | .08 | .07 |  | .11 | 18* | .13 | .09 |  | .31*** | .31*** | .13 |
| (8) |  |  |  |  |  |  |  |  |  | .84*** |  | .06 | .06 | .06 |  | 07 | .17* | .18* | .11 |  | .29*** | .23** | .15* |
| (9) |  |  |  |  |  |  |  |  |  |  |  | .03 | .02 | .05 |  | .07 | .13 | .14 | .08 |  | .27*** | .25*** | .15* |
| (10) |  |  |  |  |  |  |  |  |  |  |  |  | .72*** | .67*** |  | 0.570 | .44*** | .47*** | .43*** |  | .35*** | .41*** | .50*** |
| (11) |  |  |  |  |  |  |  |  |  |  |  |  |  | .82*** |  | .45*** | .43*** | .49*** | .41*** |  | .35*** | .41*** | .44*** |
| (12) |  |  |  |  |  |  |  |  |  |  |  |  |  |  |  | .53*** | .44*** | .42*** | .40*** |  | .36*** | .41*** | .50*** |
| (13) |  |  |  |  |  |  |  |  |  |  |  |  |  |  |  |  | .65*** | .54*** | .58*** |  | .49*** | .46*** | .54*** |
| (14) |  |  |  |  |  |  |  |  |  |  |  |  |  |  |  |  |  | .70*** | .71*** |  | .55*** | .49*** | .45*** |
| (15) |  |  |  |  |  |  |  |  |  |  |  |  |  |  |  |  |  |  | .83*** |  | .56*** | .49*** | .55*** |
| (16) |  |  |  |  |  |  |  |  |  |  |  |  |  |  |  |  |  |  |  |  | .49*** | .46*** | .48*** |
| (17) |  |  |  |  |  |  |  |  |  |  |  |  |  |  |  |  |  |  |  |  |  | .62*** | .53*** |
| (18) |  |  |  |  |  |  |  |  |  |  |  |  |  |  |  |  |  |  |  |  |  |  | .63*** |

*Notes*. The item stem for items 1-9 is “I would encourage my child to nap if…” and the stem for items 10-19 is “I would discourage my child from napping if…”;

* *p* < .05, ** *p* < .01, *** *p* < .001. *r*’s greater than|.16| are significant at *p* < .05.

**Table S5b.** Inter-item Pearson correlations for the Reasons Children Nap Scale in the Replication Sample

|  | Encouragement Reasons | | | | | | | | | |  | Discouragement Reasons | | | | | | | | | | | |
| --- | --- | --- | --- | --- | --- | --- | --- | --- | --- | --- | --- | --- | --- | --- | --- | --- | --- | --- | --- | --- | --- | --- | --- |
|  | ***Child-related*** | | | | |  | ***Parent-related*** | | | |  | ***Child-Preference*** | | |  | ***Child-Function*** | | | |  | ***Scheduling*** | | |
|  | **(1)**  I knew my child would have to stay up late tonight | **(2)**  My child had poor sleep the night before | **(3)**  My child told me they wanted a nap | **(4)** Napping was part of my child’s routine | **(5)**  My child was cranky |  | **(6)**  I needed free time | **(7)**  I needed time to do other things e.g., chores, relax | **(8)**  The timing worked for me | **(9)**  I needed a break |  | **(10)**  My child did not seem to enjoy napping | **(11)**  My child refused to nap | **(12)**  My child did not want to nap |  | **(13)**  My child slept too much the night before | **(14)**  My child got enough sleep the night before | **(15)**  My child was in a good mood | **(16)**  My child was alert |  | **(17)**  I wanted my child to have an earlier bedtime that night | **(18)** There was not enough time for a nap | **(19)** Napping would delay the time my child fell asleep at night |
| (1) |  | .52*** | .30*** | .19*** | .44*** |  | .19*** | .20*** | .30*** | .21*** |  | .15** | .17*** | .14** |  | .23*** | .20*** | .10* | .27*** |  | .24*** | .30*** | .27*** |
| (2) |  |  | .35*** | .23*** | .49*** |  | .13** | .13** | .22*** | .17*** |  | .12** | .13** | .10* |  | .17*** | .08* | -.01 | .14** |  | .16*** | .21*** | .15** |
| (3) |  |  |  | .30*** | .29*** |  | -.08* | -.02 | .09* | -.03 |  | .09* | .11* | .10* |  | .02 | .02 | -.07 | .05 |  | .01 | .07 | .04 |
| (4) |  |  |  |  | .27*** |  | .09* | .17*** | .20*** | .21*** |  | -.02 | -.06 | -.08* |  | -.01 | -.10* | -.15** | -.03 |  | -.03 | .04 | -.06 |
| (5) |  |  |  |  |  |  | .10* | .15** | .27*** | .11* |  | .06 | .09* | .03 |  | .03 | -.02 | -.13** | .10* |  | .06 | .14** | .09* |
| (6) |  |  |  |  |  |  |  | .74*** | .57*** | .74*** |  | .04 | .02 | .00 |  | .23*** | .20*** | .30*** | .15** |  | .27*** | .21*** | .18*** |
| (7) |  |  |  |  |  |  |  |  | .62*** | .76*** |  | .04 | -.02 | -.03 |  | .19*** | .21*** | .25*** | .16*** |  | .23*** | .17*** | .16*** |
| (8) |  |  |  |  |  |  |  |  |  | .59*** |  | .06 | .02 | .02 |  | .19*** | .24*** | .19*** | .19*** |  | .26*** | .19*** | .19*** |
| (9) |  |  |  |  |  |  |  |  |  |  |  | .08 | .02 | -.01 |  | .22*** | .19*** | .29*** | .18*** |  | .25*** | .21*** | .15** |
| (10) |  |  |  |  |  |  |  |  |  |  |  |  | .62*** | .61*** |  | .49*** | .47*** | .42*** | .54*** |  | .36*** | .44*** | .49*** |
| (11) |  |  |  |  |  |  |  |  |  |  |  |  |  | .77*** |  | .41*** | .39*** | .30*** | .49*** |  | .32*** | .45*** | .48*** |
| (12) |  |  |  |  |  |  |  |  |  |  |  |  |  |  |  | .44*** | .42*** | .29*** | .49*** |  | .32*** | .44*** | .52*** |
| (13) |  |  |  |  |  |  |  |  |  |  |  |  |  |  |  |  | .64*** | .53*** | .62*** |  | .59*** | .53*** | .56*** |
| (14) |  |  |  |  |  |  |  |  |  |  |  |  |  |  |  |  |  | .55*** | .62*** |  | .57*** | .47*** | .51*** |
| (15) |  |  |  |  |  |  |  |  |  |  |  |  |  |  |  |  |  |  | .50*** |  | .44*** | .33*** | .39*** |
| (16) |  |  |  |  |  |  |  |  |  |  |  |  |  |  |  |  |  |  |  |  | .50*** | .51*** | .52*** |
| (17) |  |  |  |  |  |  |  |  |  |  |  |  |  |  |  |  |  |  |  |  |  | .49*** | .58*** |
| (18) |  |  |  |  |  |  |  |  |  |  |  |  |  |  |  |  |  |  |  |  |  |  | .57*** |

*Notes*. The item stem for items 1-9 is “I would encourage my child to nap if…” and the stem for items 10-19 is “I would discourage my child from napping if…”; *

*p* < .05, ** *p* < .01, *** *p* < .001. *r*’s greater than |.075| are significant at *p* < .05.

**Table S6a.** Post-hoc differences between Parent’s Nap Beliefs Scale, Reasons Children Nap Scale, and Napping Frequency Groups in the Pilot Sample

| Napping Frequency | Did not nap in past month & Naps <1 day/week | Naps 1-3 days/week | Naps 4-5 days/week | Naps 6-7 days/week |
| --- | --- | --- | --- | --- |
|  | *n* = 69 | *n* = 39 | *n* = 28 | *n* = 63 |
| Subscale | *M* (*SD*) | *M* (*SD*) | *M* (*SD*) | *M* (*SD*) |
| **Parents’ Nap Beliefs** |  |  |  |  |
| Positive Beliefs | 2.97 (1.02) ^B, C, D^ | 3.86 (0.73) ^a, D^ | 3.98 (0.71) ^A^ | 4.21 (0.67) ^A, B^ |
| Negative Beliefs | 3.60 (0.80) ^C, D^ | 3.21 (1.03) ^B^ | 3.01 (1.22) ^A, D^ | 2.43 (1.08) ^A, B, C^ |
| **Reasons Children Nap** |  |  |  |  |
| **Encouragement** |  |  |  |  |
| Child-Related | 3.22 (1.04) ^B, C, D^ | 3.77 (0.75) ^A, D^ | 4.23 (0.7) ^A^ | 4.26 (0.7) ^A, B^ |
| Parent-Related | 2.43 (1.27) ^B^ | 3.13 (1.13) ^A^ | 2.92 (1.47) | 2.74 (1.29) |
| **Discouragement** |  |  |  |  |
| Child-Preference | 3.58 (1.05) ^D^ | 3.13 (1.1) | 3.48 (1.05) | 3.02 (1.25) ^A^ |
| Child-Function | 3.49 (1.08) ^D^ | 3.46 (0.83) ^D^ | 3.17 (0.92) ^D^ | 2.61 (1.18) ^A, B, C^ |
| Scheduling | 3.52 (0.94) ^D^ | 3.39 (1.03) ^D^ | 3.63 (0.94) ^D^ | 2.82 (1.15) ^A, B, C^ |

Note. p-values were adjusted using the False Discovery Rate.

^A^ denoted a significant difference (*p* < .05) from the “did not nap in past month & naps <1 day/week” group

^B^ denoted a significant difference (p < .05) from the “Naps 1-3 days/week” group

^C^ denoted a significant difference (p < .05) from the “Naps 4-5 days/week” group

^D^ denoted a significant difference (p < .05) from the “Naps 6-7 days/week” group

**Table S6b.** Post-hoc differences between Parent’s Nap Beliefs Scale, Reasons Children Nap Scale, and Napping Frequency Groups in the Replication Sample

| Napping Frequency | Did not nap in past month & Naps <1 day/week | Naps 1-3 days/week | Naps 4-5 days/week | Naps 6-7 days/week |
| --- | --- | --- | --- | --- |
|  | n = 51 | n = 18 | n = 47 | n = 28 |
| Subscale | *M* (*SD*) | *M* (*SD*) | *M* (*SD*) | *M* (*SD*) |
| **Parents’ Nap Beliefs** |  |  |  |  |
| Positive Beliefs | 3.16 (0.90) ^B, C, D^ | 3.69 (0.82) ^A, D^ | 3.88 (0.65) ^A^ | 4.01 (0.75) ^A, B^ |
| Negative Beliefs | 3.65 (0.83) ^B, C, D^ | 2.99 (0.88) ^A, D^ | 2.82 (0.94) ^A, D^ | 2.35 (0.97) ^A, B, C^ |
| **Reasons Children Nap** |  |  |  |  |
| **Encouragement** |  |  |  |  |
| Child-Related | 3.63 (0.80) ^D^ | 3.83 (0.79) | 3.80 (0.81) | 3.85 (0.90) ^A^ |
| Parent-Related | 2.75 (1.26) ^B, C, D^ | 3.14 (1.21) ^A, C^ | 3.66 (0.97) ^A, B, D^ | 3.32 (1.15) ^A, C^ |
| **Discouragement** |  |  |  |  |
| Child-Preference | 3.57 (1.03) ^B, C, D^ | 3.12 (1.25) ^A, D^ | 3.04 (1.23) ^A, D^ | 2.58 (1.17) ^A, B, C^ |
| Child-Function | 3.22 (1.11) ^D^ | 3.03 (1.16) ^D^ | 3.2 (1.10) ^D^ | 2.44 (1.07) ^A, B, C^ |
| Scheduling | 3.48 (1.11) ^D^ | 3.28 (1.15) ^D^ | 3.42 (1.05) ^D^ | 2.73 (1.08) ^A, B, C^ |

Note. p-values were adjusted using the False Discovery Rate.

^A^ denoted a significant difference (*p* < .05) from the “did not nap in past month” group

^B^ denoted a significant difference (p < .05) from the “Naps <1/week” group

^C^ denoted a significant difference (p < .05) from the “Naps 1-4 days/week” group

^D^ denoted a significant difference (p < .05) from the “Naps 5-6 days/week” group

^E^ denoted a significant difference (p < .05) from the “Naps daily” group


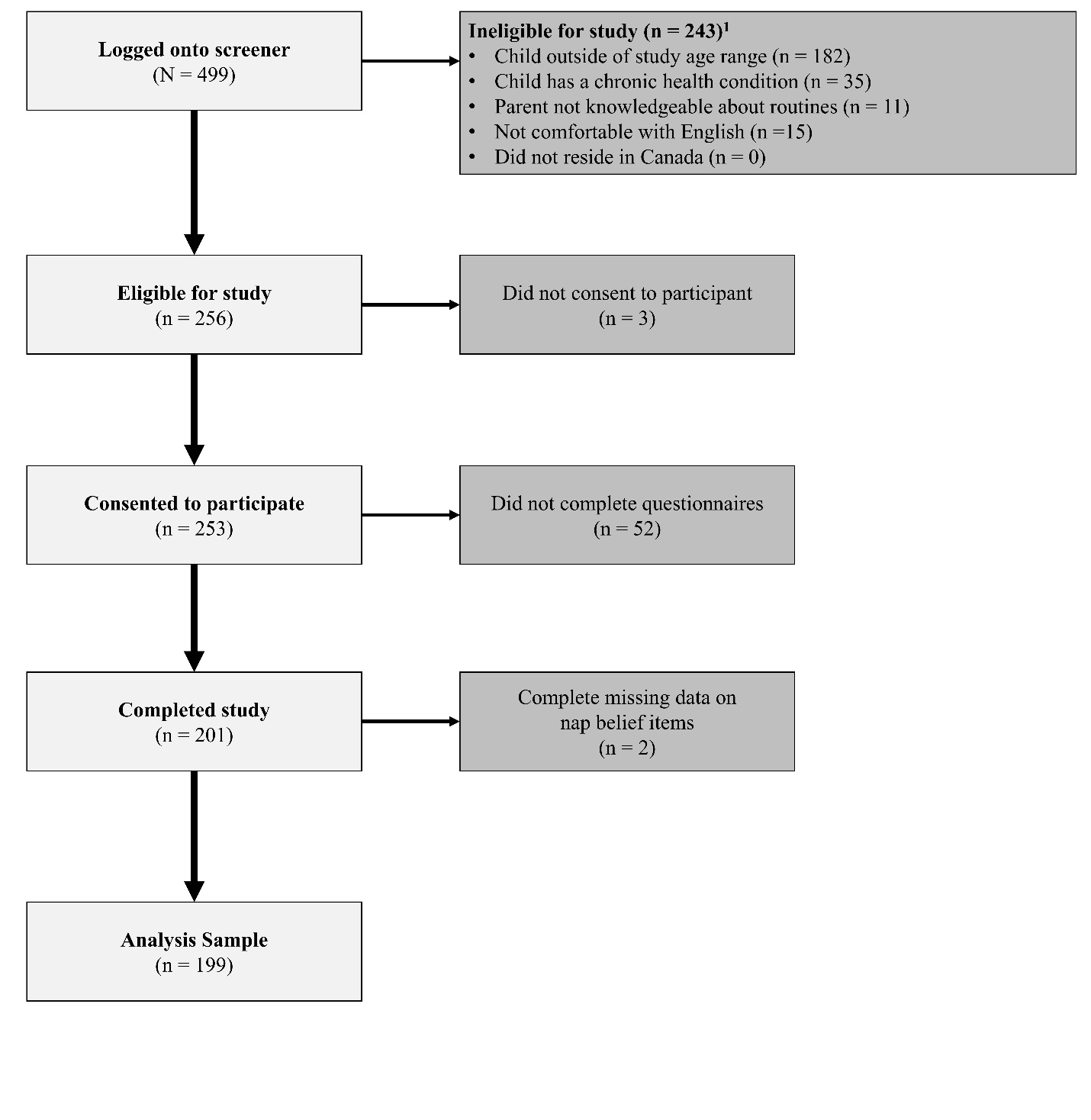


**Supplemental Figure S1a.** Participant flowchart in the Pilot Sample. ^1^ Note: exclusion criteria were sequentially ordered, such that if a parent endorsed an exclusion criterion (e.g., parent not knowledgeable about the child’s routines), they were not shown additional questions. As such, parents could not meet more than one exclusion criteria.


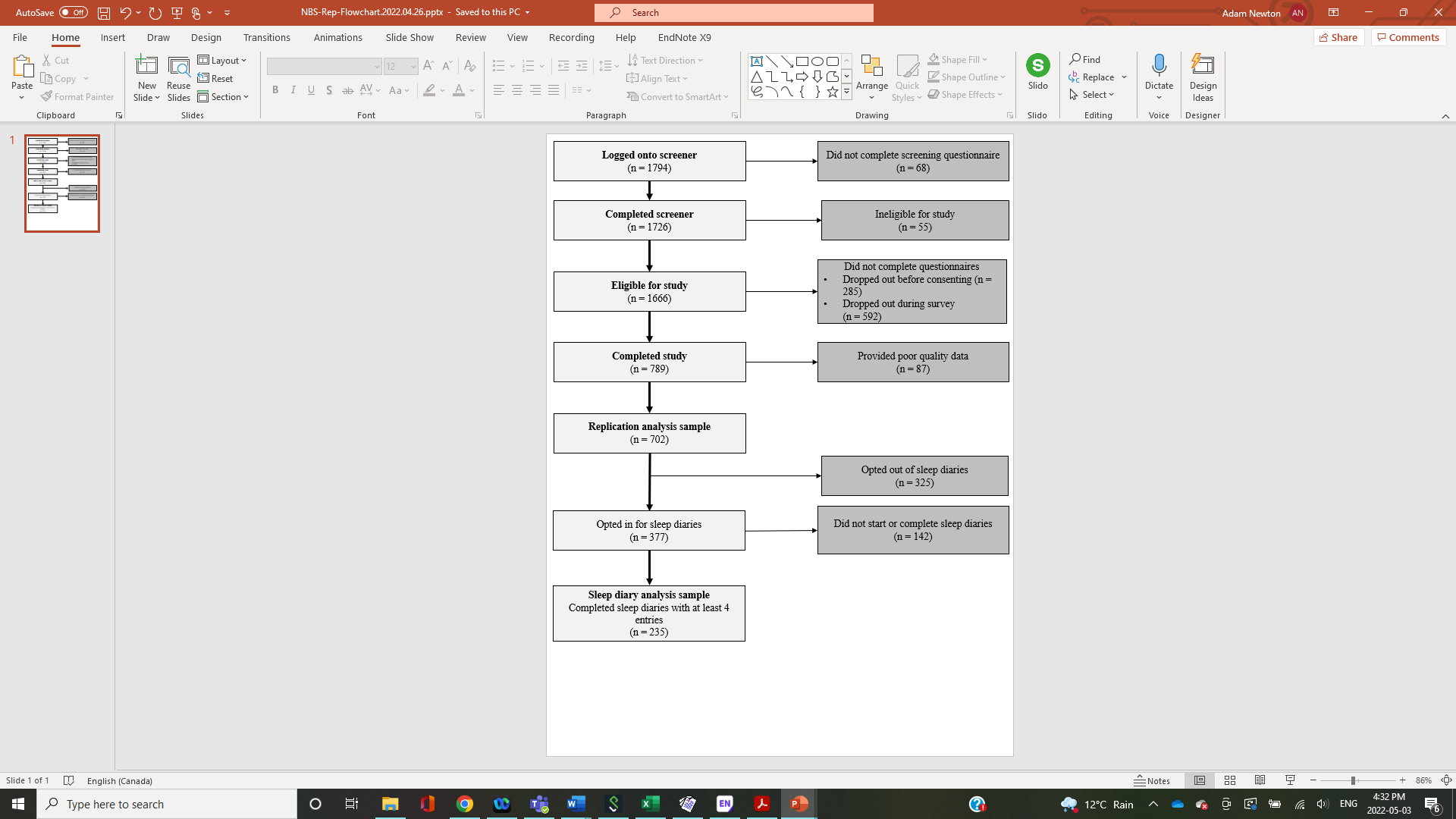


**Supplemental Figure S1b.** Participant flowchart in the Replication Sample.
